# Supplementary material for: Risk factors of child mortality in Ethiopia: Application of multilevel two-part model
Source: PLoS One. 2020 Aug 17;15(8):e0237640. doi: 10.1371/journal.pone.0237640 (PMC7430735; doi:10.1371/journal.pone.0237640)
Supplement: Attachment — Submitted filename: PLOS ONE Child mortality in Ethiopia Multilevel analysis.docx [file pone.0237640.s001.docx]

PLOS ONE

Risk Factors of Child Mortality in Ethiopia: Application of Multilevel Two-Part Model

Abstract

What do you mean by “being contraceptive use”: At which stage? Before or after the child’s death?

What do you mean by “primary level of educated mother”?

What is “being had working father”

There is no result in the abstract to justify “The findings indicate a significant regional variation of child mortality in Ethiopia.”

Objective is clear

Methods

Page 4

“The women were interviewed by distributing questioners”?

What are the justifications for including the Independent Variables?

Page 5

Citations such as “The ZIP model, introduced by [17],…..” is wrong. Replace here and elsewhere as “Lambert et al. introduced the ZIP model [17],….” or “The ZIP model, introduced by Lambert et al. [17],…..”

mothers who may not be dead her child).?

Page 7

The Poisson Hurdle and Negative binomial Poisson mixed regression model is (7) and (8) respectively?

Check : distributed with mean 0 and variance 2*u* σ and 2 *w* σ ,

And 7720 of them lost 7 at least one child.?

Page 8

The subsection “**The trend of child mortality in Ethiopia (2000-2016)**” and Figure 2 are not results of the current study. These should be incorporated into the introduction or discussion section.

Table 1

Delete all ‘%’ in the Table except in the headings

The column “No(%)” is redundant. Delete

What is p-value of <,0.001? delete the comma sign

The study population is not very clear.

I presume this study is about all deaths among all children to a woman who had ever given birth. Or is it all deaths among children born to a woman within the 5-years preceding the survey. Which data did the author used? The women data? Birth recode data? Or children recode data?

ALL these must be clearly stated under methods and Data

More importantly, since all deaths at a particular time is been studied, which birth or child was used to classify the variables in Table 1. For instance, the variable Child Twin has single and multiple. Assuming a mother had a twins birth and had a single birth thereafter, which of the births was used to classify the twin status. Same thing applies to place of delivery, Birth order number, Currently breastfeeding, Contraceptive use, Vaccination of child, Sex of child, Number of antenatal Visits and Preceding birth interval.

Variables here should be strictly mothers characteristics. The only child characteristic of interest here sis the outcome variable which is “child status: Alive or death”

This brings another issue, at which age of the children did the authors classified a child as dead or alive, since there are multiple children per woman in some cases

Table 2

The results here are not reliable because some of the variables in the models are inappropriate for the reasons stated earlier. Addition to that list is “family size”. This is not static but changes as there are more births or deaths. How can such predict death?

I stopped the review here

General comments……..

Good study design but the authors did not pay attention to basic details

Too numerous typos, incomplete statements etc.

They had reported that 7720 women had lost at least one child. And also claimed that 7720 women had lost 7 children!!!
