# Supplementary material for: Risk factors of child mortality in Ethiopia: Application of multilevel two-part model
Source: PLoS One. 2020 Aug 17;15(8):e0237640. doi: 10.1371/journal.pone.0237640 (PMC7430735; doi:10.1371/journal.pone.0237640)
Supplement: Attachment — Submitted filename: Response to reviewers-PONE-D-19-33030.doc [file pone.0237640.s002.doc]

| **Reviewer and editor # comments and questions** | **Responses** | **Location of Response in Revised Manuscript** |
| --- | --- | --- |
| You indicated that there is only a total of 11 regions in Ethiopia and that you used all of them as the grouping variable (high-level) in the multilevel model. The small number of regions used in this study could potentially bias the results presented in Table 3. You are requested to conduct a sensitivity analysis for the negative binomial hurdle model results presented in Table 3 to determine whether or not the small group size could affect the estimates. In case you are unable to do this, an alternative will be for you to provide both 95% and 99% confidence intervals for the negative binomial hurdle model results presented in Table 3 and discuss both. | This is a very important question. We have read literatures and textbooks related to the multilevel modeling. We have got a very interesting point that “ the minimum number of the higher level is at least 50” . In this study we have only 11 regions. However, the EDHS dataset was collected from 645 enumeration areas in the country. We authors reconsider the multilevel modeling by incorporating 645 EAs (instead of 11 regions) as the second level in the analysis. | Explained and corrected |
| You should also provide regional crude U5 mortality map for Ethiopia based on the data, and also provide the regional residual (regional random effects) map based on the negative binomial hurdle model results presented in Table 3 for better understanding of the regional (spatial) distribution of crude U5 mortality rates and the residual regional effects in Ethiopia. | This is again very important point to show the hot spot of the incidence. We did and map both the crude U5 moratality rates and the residuals regional effects as figure 2 and figure 3. | Corrected |
| Why did you highlight “Eighty-eight (87.7%)” | Thank you. It was typing error | Corrected |
| Table 1: The column “No(%)” must be removed. It adds no information. Rather…Total number for each category is more informative. Replace accordingly | Very good point. | Corrected |
| Change “X2 test (p-value” to ” X2 value (p-value)” | Thank you | Changed |
| Change “(95 CI for IRR)” to “(95% CI for IRR)” | Thank you | Changed |
