# Supplementary material for: Risk factors of child mortality in Ethiopia: Application of multilevel two-part model
Source: PLoS One. 2020 Aug 17;15(8):e0237640. doi: 10.1371/journal.pone.0237640 (PMC7430735; doi:10.1371/journal.pone.0237640)
Supplement: Attachment — Submitted filename: Response to Reviewers - PONE-D-19-33030R4.doc [file pone.0237640.s003.doc]

| **Reviewer and editor # comments and questions** | **Responses** | **Location of Response in Revised Manuscript** |
| --- | --- | --- |
| Kindly remove Figure 3 from the manuscript and also note that the term is "ordinary Kriging" BUT not "ordinal kriging" as stated at page 15. The removal of Figure 3 is necessary because the ordinary Kriging require detailed explanation (estimation procedure for spatial correlation, range, etc) of this procedure in the methods section, and you must present the map of the prediction variance  and the plot of the known variograms associated with the Figure 3 in the results section for proper evaluation of the interpolated map based on the said ordinary kriging method.  All these are missing in the manuscript presently. Also note that spatial prediction is not the focus of this manuscript hence the earlier request to map only the crude mortality and the residual spatial effect | Thank you for you comments and scientific contribution | corrected |
